# Ex Vivo Machine Perfusion as a Platform for Lentiviral Gene Delivery in Rat Livers

**DOI:** 10.21203/rs.3.rs-4784505/v1

**Published:** 2024-09-13

**Authors:** Irina Filz von Reiterdank, Mohammadreza Mojoudi, Raphaela Bento, McLean Taggart, Antonia Dinicu, Gregory Wojtkiewicz, J H Coert, Aebele Mink van der Molen, Ralph Weissleder, Biju Parekkadan, Korkut Uygun

**Affiliations:** Massachusetts General Hospital, Harvard Medical School; Massachusetts General Hospital, Harvard Medical School; Massachusetts General Hospital, Harvard Medical School; Massachusetts General Hospital, Harvard Medical School; Massachusetts General Hospital, Harvard Medical School; Massachusetts General Hospital, Harvard Medical School; University Medical Center Utrecht; University Medical Center Utrecht; Massachusetts General Hospital; Rutgers University; Massachusetts General Hospital, Harvard Medical School

## Abstract

Developing new strategies for local monitoring and delivery of immunosuppression is critical to making allografts safer and more accessible. Ex vivo genetic modification of grafts using machine perfusion presents a promising approach to improve graft function and modulate immune responses while minimizing risks of off-target effects and systemic immunogenicity in vivo. This proof-of-concept study demonstrates the feasibility of using normothermic machine perfusion (NMP) to mimic in vitro conditions for effective gene delivery. In this study, lentiviral vectors carrying biosensor constructs with Gaussia Luciferase (GLuc) were introduced to rodent livers during a 72-hour perfusion period, with a targeted delivery of 3 × 10^7^ infection units (IU). Following the initial 24-hour exposure required for viral transduction, an additional 48 hours was necessary to observe gene expression, analogous to in vitro benchmarks. The perfused livers displayed significantly increased luminescence compared to controls, illustrating successful genetic modification. These findings validate the ex vivo use of lentiviral particles in a rodent liver model and lay the groundwork for a broad range of applications through genetic manipulation of organ systems. Future studies will focus on refining this technology to enhance precision in gene expression and explore its implications for clinical transplantation.

## Introduction

Gene therapy is emerging as a transformative treatment strategy, offering potential cures for a diverse range of diseases by correcting underlying genetic defects (1). Its application in liver diseases is particularly promising due to the liver’s central role in metabolism. However, gene therapy faces significant challenges, including the precise targeting of specific tissues or organs without off-target effects(2, 3) and the immunogenicity caused by the viral vectors(4).

In the context of liver disease, when conventional treatment falls short, transplantation remains the only definitive solution for end-stage liver disease. However, liver transplantation is limited by a significant gap between the recipient waiting list and qualifying donors. Furthermore, post-transplant complications most often associated with ischemia-reperfusion injury (IRI) and immunological rejection are known to contribute to increased early graft dysfunction (EAD), graft loss, and mortality rates (5). Normothermic machine perfusion (NMP) offers a novel and promising method to mitigate some technical transplantation challenges. By maintaining donor organs in a near-physiological state, NMP minimizes ischemia duration, and optimizes donor-recipient matching through prolonging preservation compared to the current clinical standard of static cold storage (SCS) (6, 7). Furthermore, NMP provides a unique platform that allows for isolated exposure of the liver to therapeutic agents, effectively addressing gene therapy’s targeting challenges and reducing concerns over systemic immunogenicity. This capability not only broadens the donor pool but also facilitates the potential of auto-transplantation by enabling genetic corrections in isolated organs, thereby enhancing the overall safety and efficacy of transplantation. With hepatic allograft rejection remaining a leading cause of late graft failure, accurate and timely evaluation of long-term liver function and immunogenic state remains an important challenge to overcome. Current clinical methods primarily involve detection of symptoms of ongoing graft rejection through liver function tests and invasive biopsies (8). Considering the increasing use of marginal organs due to novel preservation techniques, these approaches often fail to provide sufficient prognostic insight at the molecular level, leaving a very short window for effective medical intervention. Consequently, genetic engineering of transplanted organs has been proposed as a promising alternative approach for early detection and modulation of rejection (9).

Despite the difficulties of translating current genetic manipulation tools to the whole-organ level, genetic engineering has become an expanding area in organ transplantation, with several gene therapy techniques using viral and non-viral delivery methods being employed to address post-transplant complications (10, 11). Combing these therapies with modern advances in the field of organ preservation, previous studies have used machine perfusion as a platform to achieve apoptosis-targeted siRNA gene silencing (12), induction of viral resistance by miRNA inhibition (13) and gene delivery through Adeno-associated virus (AAV) vectors (14), all of which provide temporary solutions limited by their short-acting mechanisms of action. More recent efforts have demonstrated success in applying the technique to immunomodulation, for example by engrafting biosensor cells into rat livers for immune monitoring following transplantation (15). However, the application of vectors with long-term stability of transduction and gene expression through integration into the host genome, such as lentiviruses, remains critical clinical significance, largely unexplored by the current literature.

To demonstrate the long-term feasibility and sustainability of this concept at the whole organ level, this study develops a protocol for 72-hour *ex vivo* NMP of rodent livers aiming to genetically modify hepatic cells through lentiviral transduction to express the bioluminescent reporter, GLuc and red fluorescent protein (mRFP), allowing for region-specific analysis of transduction and gene expression. Thereby, we investigate the potential of NMP as a platform for genetic engineering with long-term expression, with the future aim of enhancing liver transplant outcomes and addressing genetic liver diseases.

## Materials and Methods

### Production of Lentiviral Vectors

Self-inactivating lentiviral vectors were generated using triple-transfection methods in adherent human embryonic kidney 293T cells (16). Briefly, HEK293T cells were expanded in DMEM/F-12 media (Thermo Fisher Scientific) supplemented with 10% v/v FBS and 1% v/v antibiotic-antimycotic solution (Thermo Fisher Scientific). Cells were seeded at 40% confluency the day before transfection. HEK293T cells were co-transfected with pLV-EF1a-GLuc-IRES-mRFP plasmid, purchased and sequence-verified by vendor (Vector Builder Inc, Chicago, IL, USA), and two packaging plasmids, psPAX2 - plasmid #12260 (Addgene, Watertown, MA, USA) and pMD2.G - plasmid #12259 (Addgene), at a molar ratio of 3:2:1. Transfection reagent polyethylenimine (Polyplus, New York, NY, USA) was also used for enhanced transfection efficiencies. Transfection culture was carried out for 72 h until supernatant was collected, centrifuged, filtered through a 0.45 mm PES membrane filter, and stored at −80°C. Vector titer was determined via qPCR using a Lentiviral titration kit (Applied Biologic Materials, Richmond, BC, Canada) on Quant Studio 3 (Thermo Fisher Scientific). Quality control assays were carried out for determination of transduction efficiencies (TE). Increasing doses of viral particles were added to HEK293T cells along with 25mg/mL of transduction reagent Protamine Sulfate. After 72 hours, TE was determined based on GLuc secretion in the supernatant and mRFP fluorescence. Lentiviral batches with transduction efficiencies > 40% were deemed acceptable for use in liver perfusions. GLuc was selected due to its sensitive and reliable reporting as a secreted biomarker ex vivo^17^ as well as in vivo(17).

### Lentiviral Transduction in vitro

HEK-293T cells were cultured in Dulbecco’s modified Eagle’s medium (DMEM) medium (Thermo Fisher) supplemented with 10% FBS (Gibco, Life Technologies) and 1% antibiotic/antifungal v/v (Thermo Fisher). 3×105 cells were plated in 48-well plates and lentiviral vectors expressing EF1a-GLuc-IRES-RFP were added at increasing multiplicity of infections (MOIs) (20, 30, 50 and 100 IU/cell), along with 25mg/mL of transduction reagent Protamine Sulfate (Thermo Fisher). After 72h, transduction efficiency was assessed via RFP fluorescence using Celigo Imaging Cytometer (Nexcelom Bioscience) and GLuc concentration in the supernatant. Group with highest transduction efficiency (% RFP + cells) was expanded expanded and a cell bank of engineered cells was created.

### Validation of Viral Construct in vitro

Engineered cells were seeded at increasing densities and monitored over time. Supernatant samples were collected at 24-, 48-, 72- and 96-hours post-seeding and assayed for GLuc accumulation. Cells were also imaged for RFP expression.

### Experimental Design

**Figure 1:**
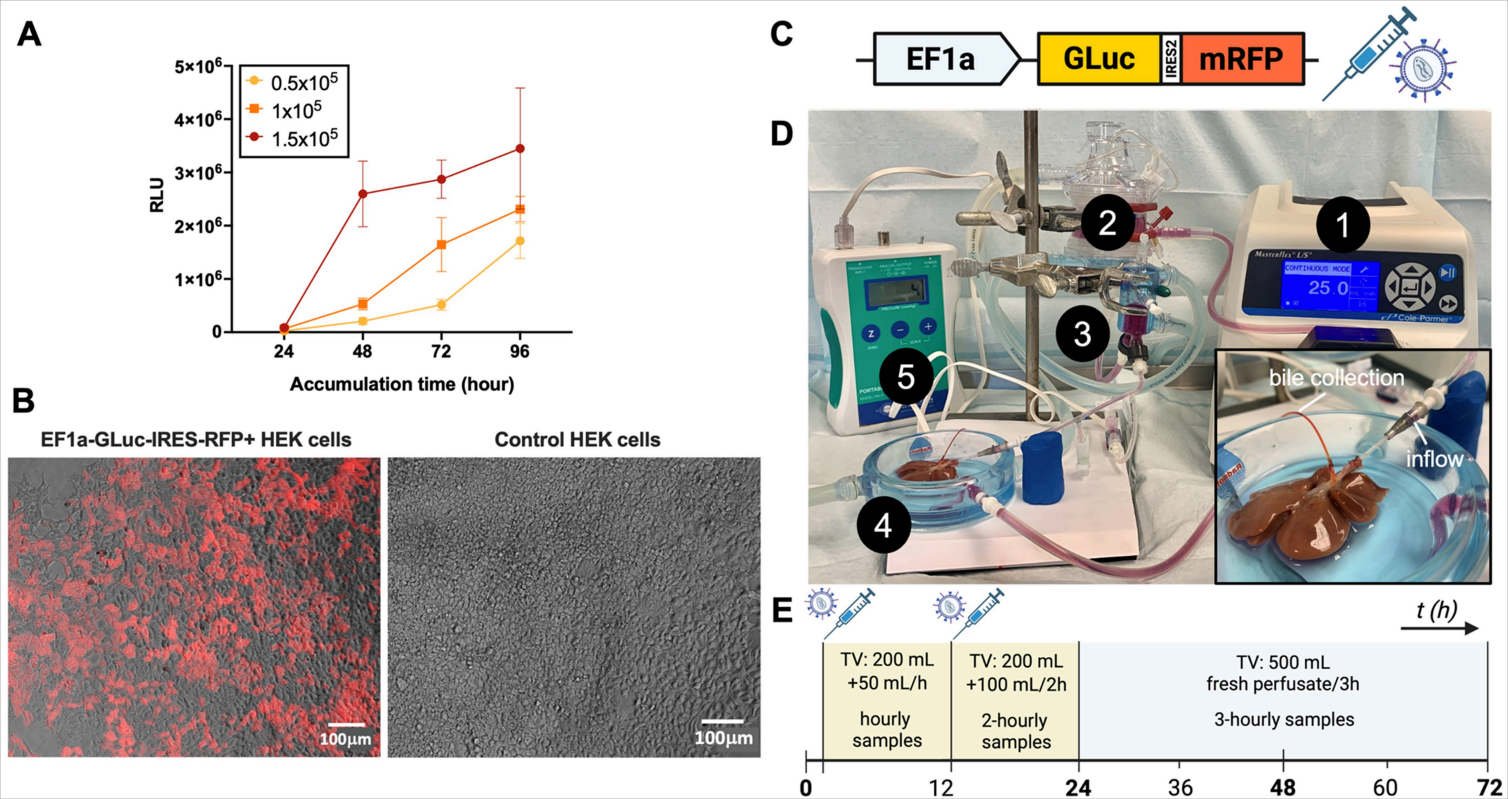
In vitro validation of lentiviral genetic construct shows time- and dose-dependent gene expression. (**A**) Engineered HEK293T cells were seeded at increasing densities and supernatant was collected over different accumulation times for GLuc assessment, which shows clear time- and dose-dependent secretion of the biomarker. (**B**) Representative images are shown of i. Engineered HEK293T cells, constitutively expressing RFP, and ii. Non-engineered HEK293T cells, which show no RFP expression. (**C**) Schematic illustration of genetic construct used (EF1a-GLuc-IRES2-mRFP) (**D**) Perfusion setup consisting of: 1. Digital Peristaltic Pump 2. Oxygenation chamber 3. Bubble trap 4. Perfusion basin 5. Pressure monitor. The image of the liver shows how the organ is connected to the perfusion system and the bile collection tube. Basin was drained of perfusate for imaging purposes. (**E**) Perfusion timeline with respect to viral exposure and removal, as well as perfusate addition and replacement. Error bars are shown as mean with range. *TV total volume*.

Whole livers were procured from adult Lewis rats and underwent one of the following conditions: (1) Control: 72-hour normothermic machine perfusion (NMP) of whole liver with no viral treatment (n = 3). (2) Viral treatment: 72-hour NMP of whole liver with exposure to lentivirus containing the genetic construct outlined in Fig. 1 (n = 4). Mimicking *in vitro* metrics (18), viral exposure lasted for 24h and transduction assessment occurred 48h after viral exposure (at 72h).

### Perfusate Preparation

Recovery perfusate was composed from a base of 500 mL Williams’ Medium E (with sodium bicarbonate, without L-glutamine, without phenol red) (Sigma-Aldrich, St. Louis, MO, USA) into which 285.7143 nM polyethylene glycol (PEG) 35 000 kDa (Sigma-Aldrich, 81310), 61.1527 μM dexamethasone (water-soluble) (Sigma-Aldrich, D4902), 150.5344 μM bovine serum albumin (Sigma-Aldrich, A7906), 9.5227 mM sodium bicarbonate (Sigma-Aldrich, S6014), 4.998 mM L-glutathione (Sigma-Aldrich, G4251), 0.01 U/mL insulin (Eli Lilly and Company, Cambridge, MA, USA, 0002821501), 10 U/mL sodium heparin (Pfizer Inc, NYC, NY, USA, 004092720), 25 μg/mL hydrocortisone sodium succinate (Pfizer Inc, 00009001305), 2 mM L-glutamine (Thermo Fisher Scientific, Waltham, MA, USA, 25030081) and 5% Antibiotic-Antimycotic solution (Thermo Fisher Scientific, 15240096) were added; L-glutamine, insulin, hydrocortisone and L-glutathione were all added to the solution no more than 24 hours prior to use.

## Liver Procurement

10–12 week-old male Lewis rats in the weight range of 250–300g (Charles River Laboratories, Wilmington, MA, USA) were used for all experiments to ensure comparable results between groups. All animals were socially housed in controlled, standard conditions (21°C, 12-hour light/dark cycle, 30–70% humidity, mixed paper/cellulose bedding, pathogen-free HEPA filtered ventilated cages). The rats had unrestricted access to sterile water and chow, in accordance with National Research Council Guidelines. Rats were cared for the Massachusetts General Hospital (MGH) Center for Comparative Medicine (CCM). The experimental protocol was approved by the Institutional Care and Use Committee (IACUC) of MGH (Protocol Number 2021N000005), and all experiments were carried out in accordance with guidelines established in said protocol. Considering the prolonged duration of the experiment rendered the organ more prone to contamination, all following surgical procedures were carried out using sterile technique, consumables and solutions. Surgical procurement was performed as described earlier (19). Donor rats were anesthetized under 5% isoflurane. The abdomen was opened via a transverse abdominal incision. Ligaments connecting the superior and inferior portions of the liver were dissected and the portal vein was exposed. The gastric and splenic branches of the portal vein, as well as the hepatic artery were ligated using 6 – 0 silk. The bile duct was partially dissected, cannulated using 24g tubing, and secured with 6 – 0 silk. The inferior vena cava was heparinized with 1 U/g using a 30 gauge insulin syringe. Following 5–10 minutes of heparin circulation, the portal vein was cannulated with a 16g cannula, followed by transection of the IVC. The cannula was connected to 16g tubing attached to a 60mL syringe containing 1mL heparin in 60mL saline. The portal vein was hand flushed at 10mL/min for 4 minutes, after which the remaining connective tissue was dissected, and the liver was removed from the body cavity. Following removal, the liver was flushed with the remaining 20mL saline, immediately weighed and connected to the perfusion system, keeping warm ischemic time below 5 minutes.

### Normothermic Machine Perfusion and Viral Treatment

Perfusate circulation was carried out as described earlier (20). Using a roller pump system (Masterflex L/S, Vernon Hills, IL, USA) with two separate sets of tubing delivering perfusate into and out of the perfusion reservoir. The system was consistently kept at a temperature of 37°C via a water bath (PolyScience, Niles, IL, USA) continuously pumping heated water through the double-jacketed perfusion system components (Radnoti, Covina, CA, USA). Perfusate oxygen concentration was maintained within a close range of 500 mmHg using a 95% O2/5% CO2 gas cylinder (Airgas, Radnor, PA, USA). System pressure was zeroed, the liver was placed in the tissue bath and connected to the system. Flow rate was brought from 5mL/min to 30mL/min gradually, maintaining a maximum portal pressure of 11mmHg and minimum flow rate of 20mL/min throughout the perfusion. In accordance with the timeline outlined in the study design (Fig. 1), outflow samples were collected directly from the basin in close proximity of the inferior vena cava, while inflow samples were collected from a port placed above the cannula perfusing the portal vein. 3-hourly replacements of perfusate following the first 24 hours of perfusion were carried out in a gradual fashion, perfusing the liver with incremental combinations of new and old perfusate to prevent potential damage. The liver was weighed following upon the end of perfusion to determine weight change. Biopsies of the left lateral lobe (LLL) and right medial lobe (RML) were carried out immediately after, with the LLL sample being snap frozen in liquid nitrogen for subsequent ATP analysis and the RML sample being formalin-fixed for histological analysis. The liver was then stored in cold saline and transported for imaging.

### Liver Viability Assessment

According to the time points established in the study design (Fig. 1), inflow and outflow perfusate samples were analyzed using a Siemens Rapidpoint 500 (Siemens, Munich, Germany). Liver performance metrics were analyzed to determine liver functionality during perfusion (pH, O2 consumption, lactate clearance, potassium). Following each experiment, collected outflow samples were analyzed for hepatic injury markers ALT and AST enzymes using a Piccolo Xpress (Abaxis, Union City, CA, USA). Portal resistance was calculated using pressure readings taken at every time point, and defined as: *pressure/flow*. *initial weight*. Oxygen consumption was defined as: *(inflow partial pressure of O*_*2*_ - *outflow partial pressure of O*_*2*_*) oxygen solubility coefficient / initial weight*. Weight change was defined as: *final weight - initial weight / initial weight*, presented as a percentage.

### Quantification of GLuc in tissue and viral RNA in perfusate

Post-perfusion, tissues were pulverized, homogenized and protein and nucleic acids were extracted from each lobe using commercial kits (Nanolight Technology, Pinetop, AZ, USA, GLuc FLASH Assay, and Qiagen, Hilden, Germany RNeasy Kit, respectively). GLuc substrate, native coelenterazine enzyme (Nanolight Technology, 303), was reconstituted at 2.7 mg/mL in 200-proof ethanol. Working solutions were made fresh, immediately prior to assay with a 1:1 000 dilution in PBS. 20ml of perfusate or tissue lysate were added to black-walled, clear-bottom 96-well plates and 100 mL of CTZ solution and immediately read using a bioluminescent plate reader (Thermo Fisher, Varioskan). All samples were read forward and in reverse to account for any time-dependent signal degradation. The concentration of GLuc in each sample was calculated by taking the average of the forward and reverse readings. Total viral RNA in perfusate samples and tissue lysates was assessed via qPCR using a lentivirus titration kit (Applied Biological Materials, Richmond, Canada, LV900).

### Bioluminescent and Fluorescent Imaging

Both the perfused liver and a freshly procured control liver were injected with 4 μg/g of liver of colentazerine prior to luminescence imaging. Bioluminescence imaging was carried out using the Spectral AMI X (Spectral Instruments Imaging Optical Imaging Platform) and Sapphire NIR fluorescent scanner (Azure Biosystems, Dublin, CA, USA) at the Center for Systems Biology Core (MGH, Boston, MA, USA) to visualize the expression of GLuc. For signal intensity analysis, background radiation was subtracted. After imaging, the liver was dissected by liver lobe and stored at −80°C for later quantitative analyses of GLuc and viral DNA.

### Histological Analysis

Liver tissue sections were stained with hematoxylin-eosin (H&E), Terminal deoxynucleotide transferase dUTP nick end labeling (TUNEL), and Periodic acid-Schiff (PAS) (Specialized Histopathology Services Core, MGH, Charlestown, MA, USA). Microscopic analysis was performed by blinded pathology assessment.

### Statistical Analysis

Statistical analysis was performed with Prism software Version 9.1.2 (Graphpad Software, San Diego, CA, USA) with a two-sided significance level of 0.05. Two-way analysis of variance (21) was performed to compare time-dependent perfusion data, followed by Tukey’s post-hoc test to examine statistical differences. Metrics were reported as means with range. Unpaired T-test was performed for the comparison of two groups in the tissue analysis, and for the comparison of three groups with no time dependence, one-way ANOVA was performed.

## Results

### Lentiviral Vector Design and Validation

Lentiviral vectors were designed with the constitutive promoter EF1a driving the expression of the secreted biomarker GLuc and the fluorescent tag RFP. *In vitro* validation of this construct showed a clear dose and time-dependent expression of both genes in HEK293-T cells, laying the foundation for the *ex vivo* liver perfusion studies (Fig. 1AC). Livers were procured with a warm ischemic time (WIT) of less than 15 minutes, ensuring minimal damage prior to perfusion. The overall average initial weight was 11.3 +/− 1.3 g, indicating uniformity in the sample set. Next, livers were connected to the machine perfusion system and perfusion was started (Fig. 1D). Livers were exposed to viral particles for 24h hours, after which they were perfused for another 48h without viral particles until 72h of perfusion was reached (Fig. 1E). Due to a technical failure, one liver reached 48h instead of 72h, results of which are shown in the biomarker analysis.

### Perfusion Parameters Show Comparable Viability Between Groups

**Figure 2:**
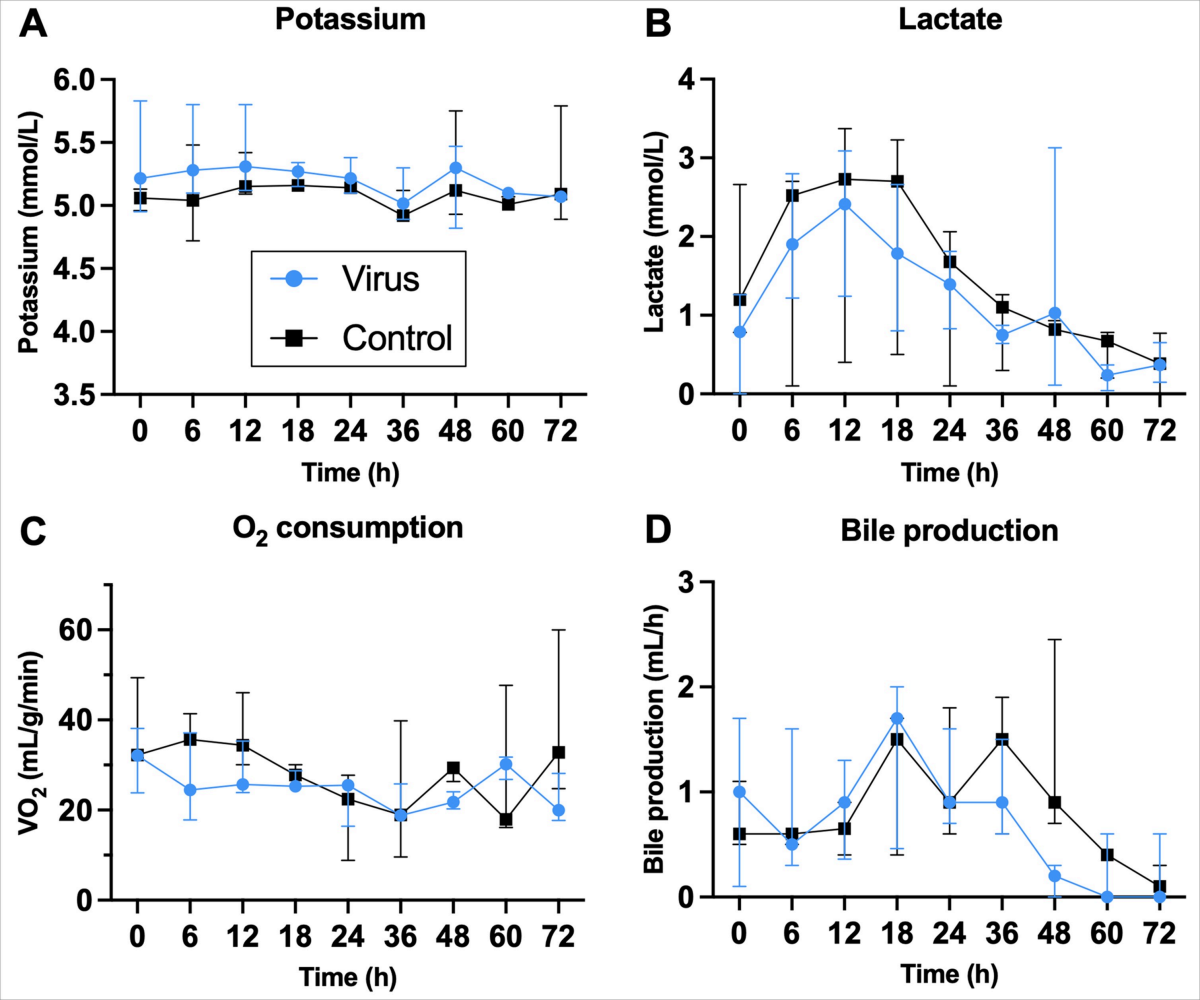
Perfusion parameters are comparable in viral and control groups. (**A**) Potassium remained stable, (**B**) lactate is cleared (**C**) and oxygen consumption remained stable throughout perfusion until end of study is reached. (**D**) Bile production shows a similar trend in both groups, tapering off towards the end of perfusion. Error bars are shown as mean with range.

Perfusion parameters were monitored to ensure stability and comparability between the experimental and control group. Prior to the viral injection time point (30 minutes), a stable flow of 30 mL/min was reached in all replicates of both the experimental and control group. Vascular resistance remained stable for 65h in all replicates and remained between 0–3 mmHg/mL/min throughout all perfusions. Further perfusion parameters are displayed in Fig. 2. Potassium remained between physiological levels of 4–6 mmol/L for both groups. Oxygen consumption remained stable throughout perfusion in both groups, suggesting effective maintenance of metabolic activity. Lactate accumulation was apparent during the first 24h to maximum levels of 3.99 mmol/L, which was the extended closed-loop phase for viral recirculation. However, clearance was apparent, especially between 12–24h in the viral group. After 24h, viral exposure was ended and perfusate switches were performed every 3 hours. During this time, lactate clearance was apparent in both groups and lasted until the end of study, indicating functional metabolic processes. Bile production lasted until at least 42h with some replicates producing bile until the end of study. Energy charge assessment indicated low but comparable levels in both groups.

### Bioluminescent and Fluorescent Imaging Demonstrate Successful Transduction

**Figure 3:**
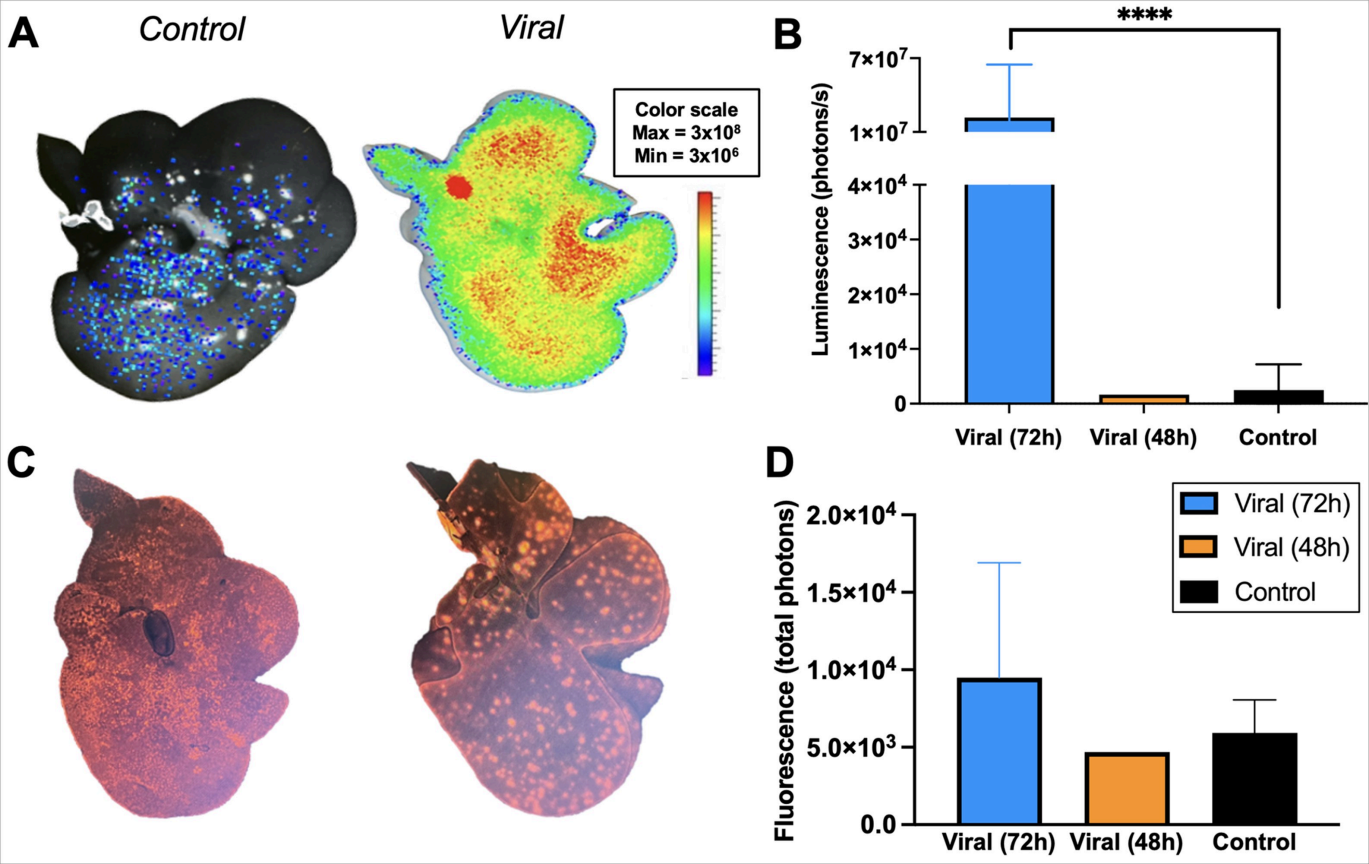
*Ex vivo*imaging show successful viral transduction post-perfusion. (**A-i**) Bioluminescent imaging shows signal intensity increase in the (33) experimental group compared to (**left**) control. (**B**) Luminescence (photons/s) are significantly higher in the experimental (viral, 72h) group versus control (unpaired T-test, p <.0001). For reference, the luminescence of the 48h viral liver is shown and demonstrates a signal comparable to the control group. (**C**) Fluorescence imaging shows hotspots that are evenly distributed throughout the liver. (**D**) While fluorescence signal intensity is higher in the viral group, the difference is not as pronounced and does not reach significance. Error bars are shown as mean with range. **** p < 0.0001.

At the end of perfusion period, livers were subjected to bioluminescent and fluorescent imaging to assess transduction success. *Ex vivo* bioluminescent imaging shows a remarkable mean 8 000-fold increase in luminescence of the transduced livers compared to controls. Signal intensity seems to be highest at the portal vein which is where the perfusate enters the liver (Fig. 3A). Furthermore, central regions, which generally are well-perfused and have higher tissue thickness, show higher signal intensity than peripheral regions. Quantitative analysis confirmed significantly higher luminescence in the experimental group (Fig. 3B, p < 0.0001). For reference, the liver that was perfused for 48h due to a technical failure is also shown and demonstrates a signal comparable to the control group. Fluorescence quantification, while lower, still shows higher fluorescence in the viral group (Fig. 3CD). These results collectively verify the effectiveness of the lentiviral vectors in achieving successful gene expression in the liver tissue.

### Biomarker Analyses Verify Transgene Expression in All Liver Lobes

**Figure 4:**
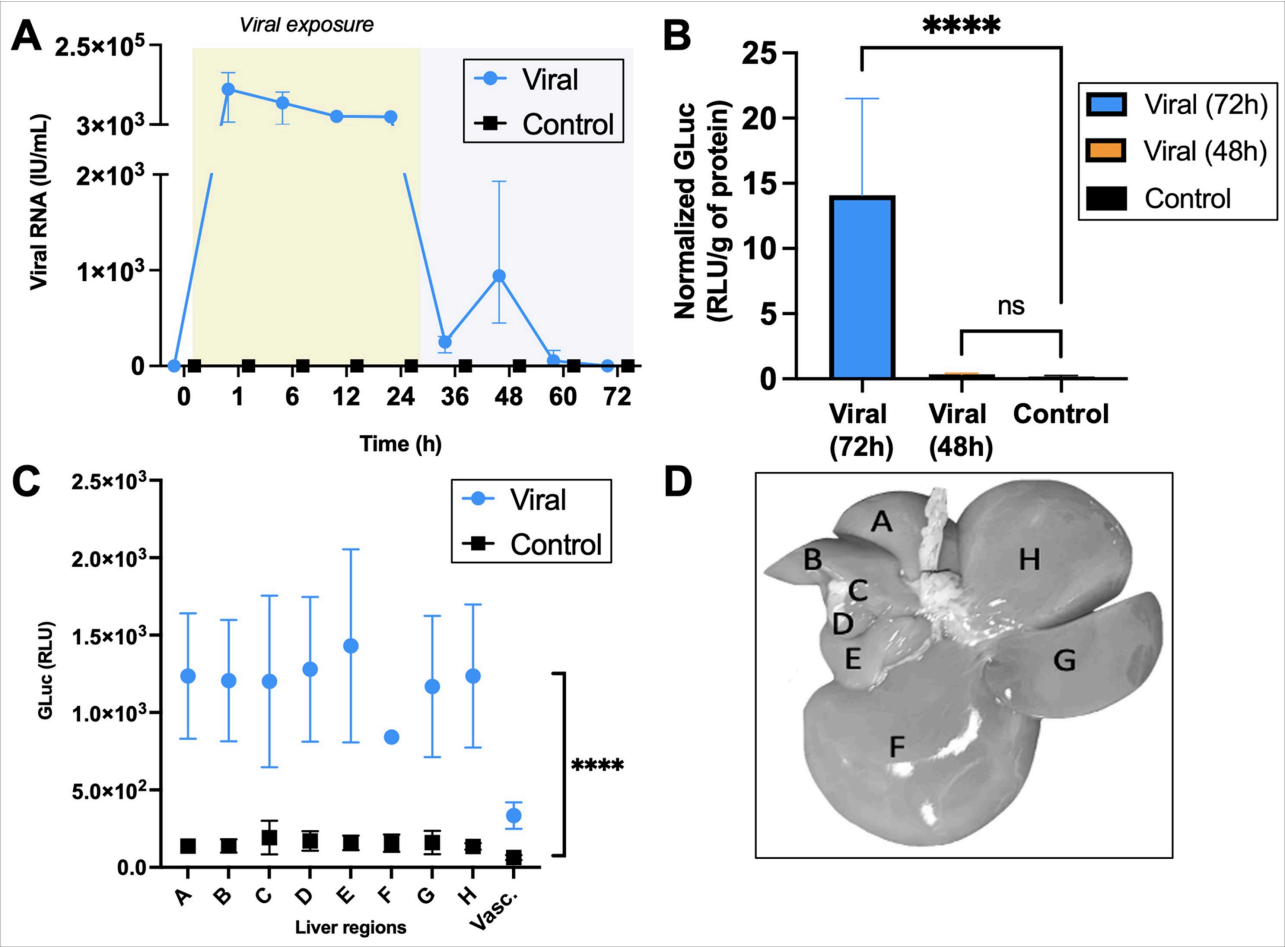
Tissue and perfusate analyses of viral RNA and GLuc show successful transduction. (**A**) Perfusate analysis for viral RNA shows successful elimination of viral particles after 24h of exposure is reached. (**B**) Tissue GLuc analysis of all liver lobes grouped per replicate (normalized against total protein) shows significant levels of GLuc in the livers exposed to viral particles that reached 72h (1-way ANOVA, p < 0.0001). Conversely, the liver exposed to viral particles that did not reach 72h but 48h showed no significant levels compared to control. (**C**) Accordingly, lobe per lobe examination shows that significant GLuc levels are reached at 72h (2-way ANOVA, column factor p < 0.0001, row factor ns). (**D**) Liver image shows which regions were selected for analysis. Error bars are shown as mean with range. **** p < 0.0001.

To further confirm transgene expression, perfusate and tissue samples were analyzed for viral RNA and GLuc presence. (Fig. 4). Perfusate analysis revealed a clear increase in viral RNA after injection of the viral particles, followed by steady decline over the first 24h suggesting uptake of the viral particles by the liver. After the perfusate switch at 24h, the viral RNA returns to pre-injection levels, suggesting successful clearance of the viral particles which persists until the end of perfusion. A similar trend was seen in the bile secretion. Tissue analysis shows a significant increase in GLuc signal at 72h (p < 0.0001). Conversely, at 48h no significant GLuc levels are found (Fig. 4B), highlighting the importance of the full 72h period for effective transgene expression. Assessment of GLuc per liver lobe shows homogenous distribution throughout the lobes with a similar column factor (p < 0.0001).

### Histological Findings Are Comparable Between Groups

**Figure 5:**
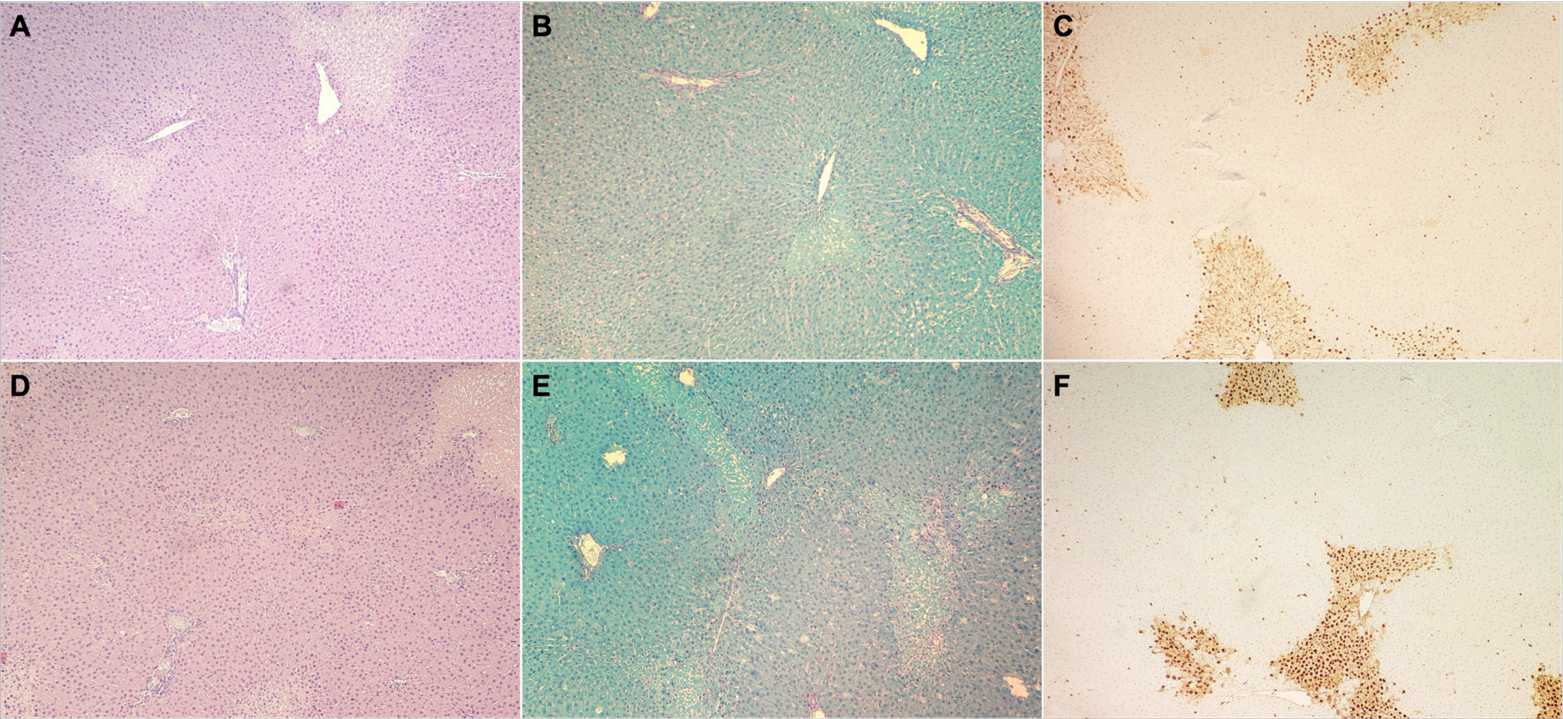
Histological analysis shows comparable results between groups. Cross-section, light microscopy, x4, Hematoxylin and Eosin (H&E) staining (**A, D**), Periodic acid-Schiff (PAS) staining (**B, E**), Terminal deoxynucleotidyl transferase dUTP nick end labeling (TUNEL) staining (**C, F**). (**A - C**) Shows viral tissue slides, (**D – F**) shows control tissue slides.

Histological analysis at the end of perfusion revealed similar levels of apoptosis between transduced and control livers. Interstitial edema and vacuolization were observed to be variable within groups but remained comparatively similar between groups (Fig. 5). These findings suggest that the lentiviral transduction process did not induce additional histological damage beyond what was observed in the control group, indicating a level of safety and feasibility for this approach.

## Discussion

This study aimed to explore the feasibility of direct genetic modification of liver grafts using lentiviral vectors, with a specific focus on the extended duration of NMP necessary for effective transduction. A perfusion period of 72h was chosen based on parallels with in vitro transduction time (22). Results demonstrated successful transduction in the livers exposed to viral particles. The time duration to expression seemed critical, as indicated by the lack of transduction observed in the replicate that underwent only 48h of perfusion before technical failure, in both tissue and imaging analysis for luminescence. This finding suggests that a wait period of at least 48h after lentiviral exposure is necessary to observe transduction.

Differential expression levels of mRFP and GLuc in our bicistronic genetic construct present an interesting aspect of this study. The weaker expression of mRFP, as compared to GLuc, is attributed to its position downstream of the internal ribosome entry site (IRES) and the inherent difficulty of measuring RFP compared to luciferase in livers. This configuration inherently limits the transcription efficiency of the downstream gene, a phenomenon consistent with observations in similar in vitro setups. In assessing the factors influencing luminescence signal intensity, associations with perfusion efficiency and tissue density were observed, resulting in greater luminescence signal in the central regions of the livers. Furthermore, detailed tissue assessment showed relative homogeneity in transduction efficiency throughout the liver lobes. These findings provide insights into the distribution and efficacy of viral transduction across liver grafts and highlight the complexity of interpreting imaging results in the context of organ perfusion and genetic modification.

A notable observation during the perfusion process in this study was the gradual decline in bile production, likely due to the absence of gall salts in the perfusion medium. This phenomenon underscores the differences between the in vitro perfusion environment and the in vivo conditions, raising important considerations for the functionality and viability of the organ during extended perfusion. In the field of preservation, many advances have been made, including perfusions of human livers up to 12 days. Research in human livers has shown successful long-term perfusion (23–25), making use of several optimizations to the perfusion system, such as automated management of oxygen and glucose levels, waste-product removal, and hematocrit control when using cellular perfusates. While this study shows perfusion of rat livers beyond current limits in literature of 24 hours (26), the viability of the liver grafts post 72-hour perfusion in this study was significantly impaired, partly due to lack of the mentioned optimizations applied to human studies. Other adaptations to address the metabolic needs of long-term perfused organs could be the use of oxygen carriers, alternative energy sources, and radical scavengers. It is important to emphasize that the primary objective of this study was to establish a proof-of-concept for the genetic modification of liver grafts using lentiviral vectors, which was successfully achieved, with potential applications extending beyond transplantation to include disease modeling and pharmacological testing. The potential for transplantation post-24-hour perfusion, along with the possibility of further reducing the required transduction time of genetic modification through optimization, remains another promising avenue for future research.

Earlier whole organ gene therapy studies in rodent livers showed engineered cell engraftment after 3-hour perfusion by injecting genetically engineered rat fibroblasts (15). However, when this was applied to transplanted vascularized composite allografts, plasma signals of the biomarker ceased after 5 days, suggesting these cells may be cleared or are not able to maintain their function after transplantation (27). Using adenoviral vectors (AAVs), a proof-of-concept study was performed showing transduced biomarker GFP through immunohistochemistry at 24h post-transplantation (14). However, no detailed RNA or DNA assessments were performed. Similar results were achieved using siRNA targeting the Fas-receptor(12, 28), again with a vector suited for temporary genetic modification. In a pig transplant study, the oligonucleotide miravirsen was used to inhibit HCV replication. Three days post-transplantation, qPCR of liver tissue showed mRNA expression and animals displayed no signs of toxicity compared to control groups (13).

The ability to genetically modify liver grafts to express markers such as GLuc and mRFP paves the way for non-invasive, prompt diagnosis of acute and chronic graft rejection, as well as other applications such as early treatment of IRI after organ preservation, with potential future uses in prolonged cryopreservation approaches. This technology may also have broader applications for the treatment of monogenic diseases, such as the correction of genetic disorders like Wilson’s disease directly within the donor liver prior to transplantation (29). Such capabilities significantly expand the therapeutic potential of liver transplants, potentially reducing or even eliminating the need for transplantation for some genetic disorders. Moreover, the successful use of NMP for gene delivery, using viral as well as non-viral vectors, sets the stage for using this technology beyond traditional transplantation applications. For instance, the platform could facilitate pharmacological testing and disease modeling, especially with advancements in maintaining human-sized livers on perfusion systems for extended periods (30). These systems could revolutionize the way we study liver diseases and test new drugs, providing a controlled, physiologically relevant environment for extended studies.

Despite promising outcomes of this study and in literature, significant challenges remain in translating these techniques into clinical practice. One of the primary barriers is ensuring the long-term stability and expression of the transgenes without risking oncogenesis. Leveraging its benefits, CRIPR-Cas9 technologies may provide solutions to challenges with permanency and precision of genetic modifications. From practical standpoint, upscaling to larger size organs will require determining vector-specific dose-dependence(31), optimal viral vector selection(30) and establishment of optimal perfusion conditions such as perfusate composition and exposure time. Regulatory and ethical considerations also play a critical role, as the use of genetically modified organs in humans requires rigorous evaluation to establish safety and efficacy standards that comply with medical regulations. In this, lessons learned from xenotransplantation could provide a useful resource (32).

In conclusion, this study shows successful lentiviral transduction of rodent livers using NMP. Furthermore, results suggest a 48-time window after viral exposure is necessary to allow for translation of the transduced genetic construct. Looking forward, the applications of our proof-of-concept study could extend well beyond the realms of rejection detection and IRI monitoring. The feasibility of using vectors for long-term gene expression in liver grafts opens up endless possibilities, ranging from modulation of inflammatory responses to enhancements in cryopreservation techniques. Thus, this study lays the groundwork for a new era in liver transplantation, where genetic engineering plays a pivotal role in improving graft outcomes and expanding the horizons of transplant medicine.

## Data Availability

All data generated and analyzed during this study have been included in this manuscript and its Supplementary Information file unless stated otherwise. All raw data can be provided upon request to the corresponding author.
